# Transcriptional Activation of Human GD3 Synthase (hST8Sia I) Gene in Curcumin-Induced Autophagy in A549 Human Lung Carcinoma Cells

**DOI:** 10.3390/ijms19071943

**Published:** 2018-07-02

**Authors:** Miri Lee, Kyoung-Sook Kim, Abekura Fukushi, Dong-Hyun Kim, Cheorl-Ho Kim, Young-Choon Lee

**Affiliations:** 1 Department of Medicinal Biotechnology, College of Health Sciences, Dong-A university, Busan 604-714, Korea; miliam9309@naver.com (M.L.); sook6624@naver.com (K.-S.K.); mose79@dau.ac.kr (D.-H.K.); 2 Molecular and Cellular Glycobiology Unit, Department of Biological Sciences, Sungkyunkwan University, Seobu-Ro, Jangan, Suwon, Gyeonggi-Do 16419, Korea; pokusa6@hotmail.com

**Keywords:** autophagic cell death, curcumin, human GD3 synthase (hST8Sia I), A549 cells, transcriptional regulation

## Abstract

Curcumin, a natural polyphenolic compound isolated from the plant *Curcuma longa*, is known to induce autophagy in various cancer cells, including lung cancer. In the present study, we also confirmed by LC3 immunofluorescence and immunoblotting analyses that curcumin triggers autophagy in the human lung adenocarcinoma A549 cell line. In parallel with autophagy induction, the gene expression of human GD3 synthase (hST8Sia I) responsible for ganglioside GD3 synthesis was markedly elevated in response to curcumin in the A549 cells. To investigate the transcriptional activation of hST8Sia I associated with the autophagy formation in curcumin-treated A549 cells, functional characterization of the 5′-flanking region of the hST8Sia I gene was carried out using the luciferase reporter assay system. Deletion analysis demonstrated that the -1146 to -646 region, which includes the putative c-Ets-1, CREB, AP-1, and NF-κB binding sites, functions as the curcumin-responsive promoter of hST8Sia I in A549 cells. The site-directed mutagenesis and chromatin immunoprecipitation assay demonstrated that the NF-κB binding site at -731 to -722 was indispensable for the curcumin-induced hST8Sia I gene expression in A549 cells. Moreover, the transcriptional activation of hST8Sia I by the curcumin A549 cells was strongly inhibited by compound C, an inhibitor of AMP-activated protein kinase (AMPK). These results suggest that curcumin controls hST8Sia I gene expression via AMPK signal pathway in A549 cells.

## 1. Introduction

Autophagy is a self-degradation process by which dysfunctional cellular components are degraded inside the cell through the process of autophagosome/autolysosome formation and autolysosomal catabolic function. This process contributes to basal cellular and tissue homeostasis [1]. Autophagy has been extensively involved in many pathophysiological processes such as cancer, metabolic and neurodegenerative disorders, and cardiovascular and pulmonary diseases [2]. Autophagy is well known to be triggered in response to a variety of biological stress stimuli such as nutrient or growth factor starvation, hypoxia, pathogen infection, DNA damage, and pharmaceutical agents [3].

Curcumin, a yellow spice, and polyphenolic compound extracted from the rhizome of the herb *Curcuma longa*, has been reported to exhibit numerous activities including antioxidant, antimicrobial, anti-inflammatory, and anticancer effects [4,5,6,7,8]. Recent studies demonstrated that curcumin also induces autophagy in various types of cancer cells, such as malignant gliomas [9], prostate cancer [10], breast cancer [11], osteosarcoma [12], gastric cancer [13], oral squamous cell carcinoma [14], uterine leiomyosarcoma [15], leukemia [16], and colon cancer [17,18]. In addition, it was recently reported that curcumin triggers autophagy in human lung adenocarcinoma cells [19,20].

Gangliosides are sialic acid-bearing glycosphingolipids (GSLs) that play vital roles in cell proliferation and differentiation, cell adhesion, and signaling processes [21,22,23,24]. Recent studies have shown that gangliosides play an important role in autophagy induction [25,26,27]. Hwang et al. demonstrated that a gangliosides mixture induces autophagic cell death in astrocytes, which is mediated by reactive oxygen species (ROS) generation, inhibition of the AKT-mTOR pathway, and activation of the the ERK pathway [25]. They also revealed that gangliosides induced-autophagic cell death in astrocytes was triggered by the IKK/NF-κB pathway [26] and GT1b might be the main active constituent of the gangliosides mixture [25,26]. Matarrese et al. also demonstrated that in human primary fibroblasts, ganglioside GD3 has a functional role in autophagosome biogenesis and/or maturation through the association of GD3 with LC3 and LAMP1, two key molecules involved in autolysosome formation and maturation [27]. However, the transcriptional regulation of the gene expression of ganglioside synthases responsible for ganglioside expression during autophagy induction has not yet been reported.

Therefore, the current study was undertaken to explore the regulatory mechanism of the gene expression of ganglioside synthases in curcumin-induced autophagy in A549 human lung carcinoma cells. In this study, we found that the mRNA expression level of hST8Sia I specifically increased simultaneously with autophagy induction by curcumin treatment in A549 cells. Furthermore, to explore the molecular basis underlying the hST8Sia I gene expression in autophagy induction by curcumin, functional characterization of the promoter region to direct the transcriptional activation of the hST8Sia I gene in curcumin-treated cells was performed.

## 2. Results

### 2.1. Effect of Curcumin on Cell Growth

**Figure 1:**
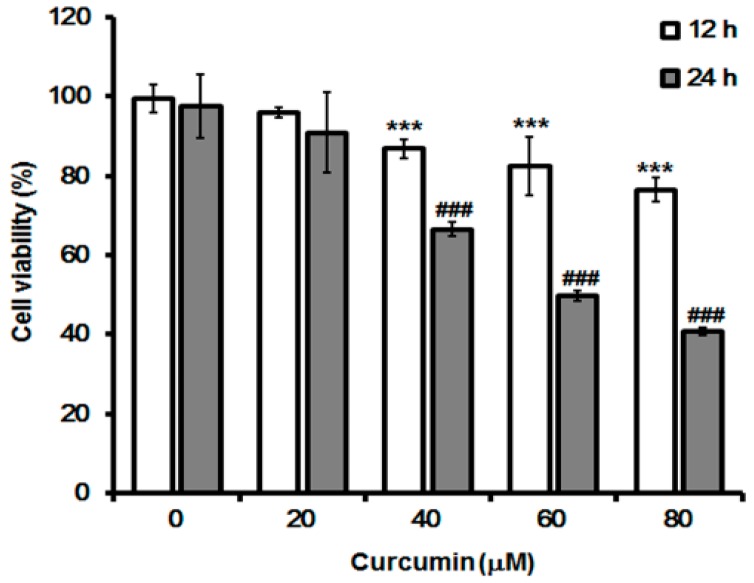
Effect of curcumin on A549 cell viability. The cytotoxic effects of curcumin on A549 cells were examined by MTT assay. Cells were cultured in growth medium at different concentrations (0–80 μM) for 12 h and 24 h, and absorbance at 540 nm was measured using an ELISA reader. Bar graphs indicate the percentage of viability. All data are expressed as mean ± SEM of three independent experiments. *** *p* < 0.0001 compared with white bar 0 μM. ### *p* < 0.0001 compared with gray bar 0 μM.

Before the effect of curcumin on autophagy induction and hST8Sia I gene expression was investigated, the growth inhibitory effect of curcumin on A549 cells was carried out by MTT assay. As shown in Figure 1, curcumin showed an inhibitory effect on cell growth in a dose- and time-dependent manner. Cell viability was significantly reduced in curcumin-treated cells for 24 h compared with those for 12 h.

### 2.2. Curcumin Induces Autophagy in A549 Cells

**Figure 2:**
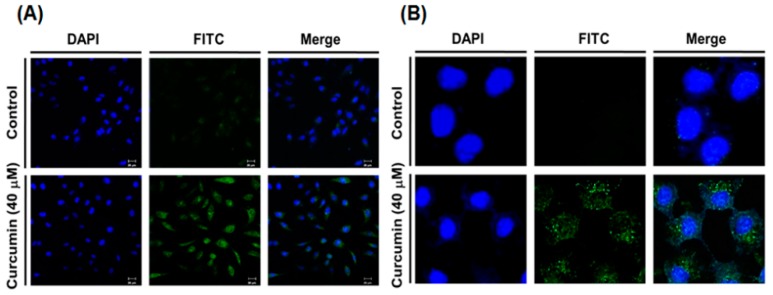
Induction of autophagy by curcumin in A549 cells. A549 cells were treated with 40 μM curcumin for 24 h and LC3 immunofluorescence staining was conducted to observe autophagosomes. DAPI staining was performed to observe the nuclei. Images were acquired using a confocal laser scanning microscope (**A**, 200×; **B**, 400×).

Previous studies showed that curcumin induced autophagy in A549 cells [19,20]. To confirm autophagy induction by curcumin in A549 cells, LC3 immunofluorescence staining was performed using fluorescent antibodies to LC3, known as specific marker of autophagosome. As shown in Figure 2, the intracellular localization of LC3 and a punctuated green fluorescence pattern of LC3 were observed in curcumin-treated A549 cells by immunofluorescence confocal microscopy, whereas curcumin-untreated control cells did not exhibit an evident fluorescence intensity, demonstrating the intracellular localization of punctate LC3.

### 2.3. Effect of Curcumin on hST8Sia I Gene Expression in A549 Cells

**Figure 3:**
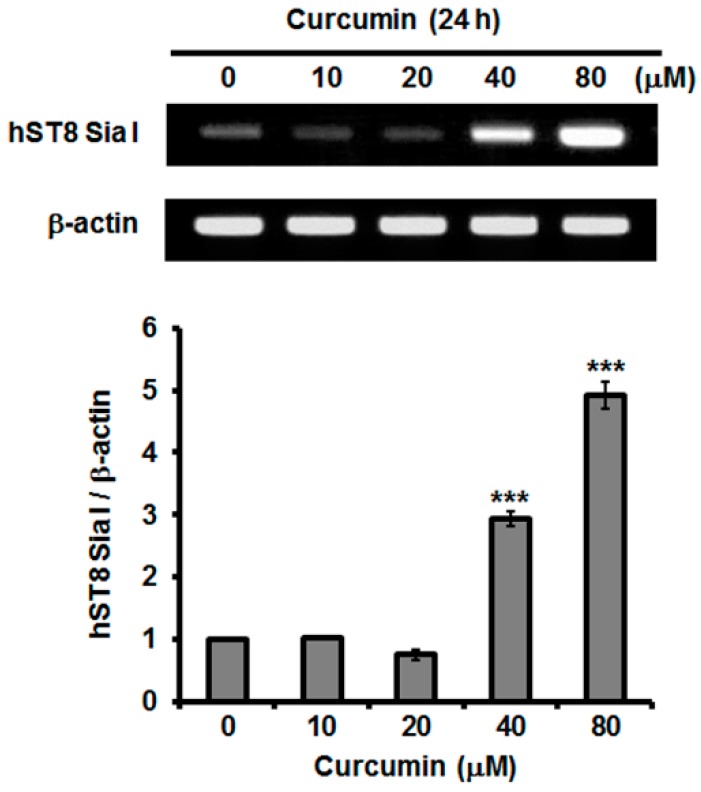
Effect of curcumin on mRNA levels of hST8Sia I. Total RNA from A549 cells was isolated after incubation at different concentrations (0–80 μM) for 24 h and mRNA level of hST8Sia I was assessed by reverse transcription polymerase chain reaction (RT-PCR). The housekeeping gene β-actin was used as an internal control. All error bars are expressed as mean ± SEM of two independent experiments. *** *p* < 0.0001 compared with 0 μM.

To examine whether autophagy induction is associated with ganglioside synthesis, we assessed the effect of curcumin on the transcription levels of human ganglioside synthase genes in A549 cells. As shown in Figure 3, the gene expression levels of hST8Sia I catalyzing ganglioside GD3 synthesis were remarkably increased in response to curcumin and their increments were in a dose-dependent manner, indicating that the induction of hST8Sia I gene expression by curcumin is controlled at the transcriptional level.

### 2.4. Effect of Curcumin on Ganglioside GD3 Expression in A549 Cells

**Figure 4:**
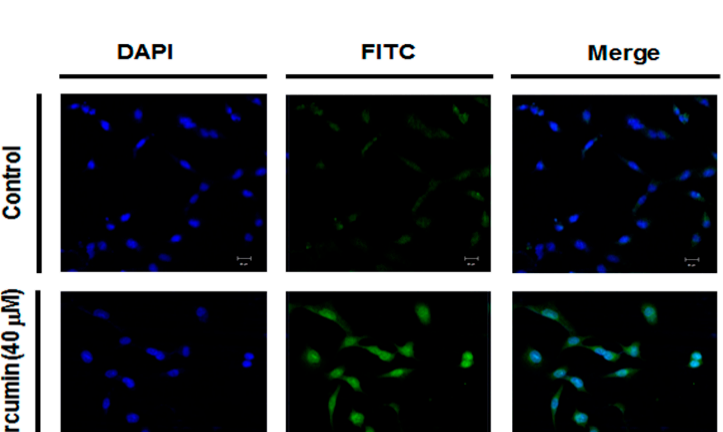
Immunofluorescence staining of ganglioside GD3 in A549 cells treated with curcumin. After curcumin treatment for 24 h, immunostaining using anti-GD3 antibodies (FITC; green) and DAPI staining (blue) were performed, and analyzed by confocal laser scanning microscope (200×).

To assess whether or not the increase of curcumin-induced hST8Sia I gene expression leads to the increment of ganglioside GD3 level synthesized by hST8Sia I in A549 cells, the cellular expression level of ganglioside GD3 was analyzed by immunofluorescence confocal microscopy using anti-GD3 mAb and fluorescein isothiocyanate (FITC)-conjugated anti-mouse IgG/M/A mixture as a secondary antibody to visualize curcumin-triggered GM3 expression in the A549 cells. As shown in Figure 4, ganglioside GD3 expression was markedly increased in the A549 cells treated with 40 mM curcumin for 24 h, but not in the curcumin-untreated control A549 cells.

### 2.5. Characterization of Curcumin-Inducible hST8Sia I Promoter in A549 Cells

**Figure 5:**
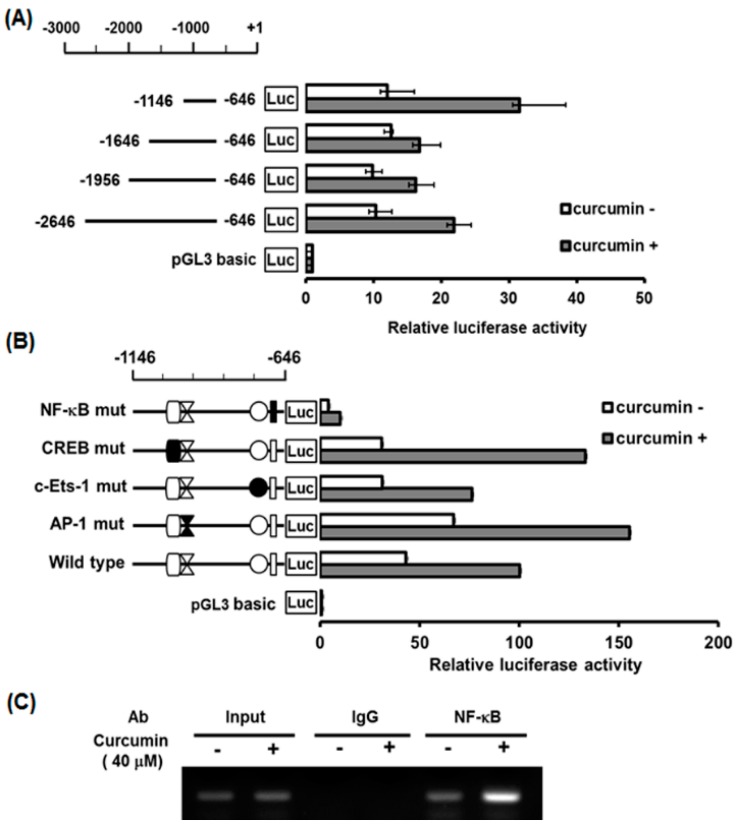
Effect of curcumin on hST8Sia I promoter activity in A549 cells. The schematic diagrams represent DNA constructs (**A**) containing 5′-deletion of the wild-type hST8Sia I promoter, or constructs (**B**) with mutants c-Ets-1, AP-1, CREB, and NF-κB sites located at the -1146 to -646 region; the start codon is designated as +1. The promoterless pGL3-basic construct was used as a negative control. Each construct and pRL-TK as an internal control were co-transfected into A549 cells. Transfected cells were cultivated in the presence (solid bar) or absence (open bar) of 40 μM curcumin for 24 h. Relative firefly luciferase activity was measured using the Dual-Luciferase Reporter Assay System, and all firefly activity was normalized to the *Renilla* luciferase activity derived from pRL-TK. (**C**) PCR amplification in the -1146 and -646 regions of the hST8Sia I promoter on immunoprecipitated chromatin obtained from A549 cells treated with or without curcumin. The input (10-fold diluted) represents the positive control. hST8Sia I mRNA was detected by RT-PCR. β-Actin was utilized as an internal control.

We then investigated whether the transcriptional activity of hST8Sia I is regulated in response to curcumin in A549 cells. Based on the significant increase of hST8Sia I gene expression in curcumin-treated A549 cells (Figure 3), the transcriptional activity of the hST8Sia I promoter was measured using the luciferase reporter assay system. In A549 cells transfected with the pGL3-1146/-646 construct, as shown in Figure 5A, the luciferase activity caused about a two-fold increase with curcumin treatment compared with curcumin-untreated cells. However, the luciferase activities of the A549 cells transfected with other promoter constructs and the pGL3-basic (negative control) did not show significant augmentation in response to curcumin stimulation. These data suggested that the region between -1146 and -646 could mediate activation of the hST8Sia I promoter by curcumin in A549 cells.

### 2.6. Identification of Curcumin-Responsive Element Controlling Inducible Expression of hST8Sia L in the Functional -1146/-646 Region of Its Promoter

Our previous studies showed that putative binding sites (for transcription factors) such as the c-Ets-1, AP-1, CREB, and NF-κB binding sites, were present in the -1146/-646 region [28,29,30,31,32]. To determine which binding site is essential in the curcumin-induced expression of hST8Sia I gene in A549 cells, luciferase assays using four mutants (pGL3-1146/-646mtCREB, mtAP-1, mtNF-κB, and mtc-Ets-1) constructed in a previous work [28,29,30,31,32] were performed. As shown in Figure 5B, of the four mutants, only pGL3-1146/-646mtNF-κB suppressed almost all of the transcriptional activity in the curcumin-treated cells. Meanwhile, the activities of the pGL3-1146/-646mtCREB and mtAP-1 mutants were significantly increased by curcumin treatment compared with wild-type pGL3-1146/-646, whereas the mtc-Ets-1 mutant was slightly reduced. These results indicated that the NF-κB binding site at positions from -731 to -722 is indispensable for the curcumin-induced expression of hST8Sia I gene in A549 cells. Next, to validate in vivo binding of NF-κB to its binding site located between -1146 and -646 of the hST8Sia I promoter in curcumin-induced A549 cells, we carried out a chromatin immunoprecipitation (ChIP) assay with antibody directed against NF-κB. As shown in Figure 5C, polymerase chain reaction (PCR) analysis using primers flanking the NF-κB binding site on the hST8Sia I promoter clearly indicated the increased amplification of the hST8Sia I promoter sequence containing the NF-κB binding site in curcumin-treated A549 cells compared with untreated cells. No amplified PCR product was observed in the ChIP assay using the nonspecific IgG antibody. Taken together, these results indicate that the hST8Sia I gene expression in the curcumin-triggered A549 cells is upregulated by the NF-κB binding to its site in the hST8Sia I promoter.

### 2.7. Curcumin Mediates hST8Sia I Gene Transcription via AMPK Pathway in A549 Cells

**Figure 6:**
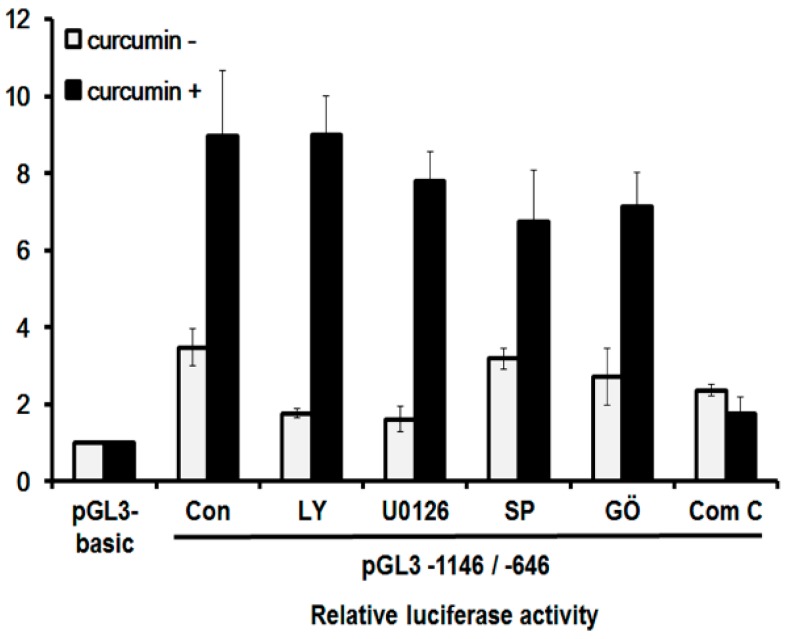
Effect of curcumin on signaling pathway for transcriptional activation of hST8Sia I A549 cells. The pGL3-1146/-646 (positive control), pGL3-basic (negative control), and pRL-TK (internal control) were co-transfected into A549 cells. Transfected cells were incubated in the presence (open bar) and absence (solid bar) of 40 μM curcumin with LY294002 (10 μM), U0126 (10 μM), SP600125 (5 μM), GO6983 (100 nM), and compound C (10 μM) inhibitors for 24 h. Relative luciferase activity was normalized with the *Renilla* luciferase activity derived from pRL-TK. The values represent mean ± SEM for three independent experiments with triplicate measurements.

Curcumin is known to induce autophagy by inhibiting the Akt/mTOR pathway and activating the ERK1/2 pathway in human malignant glioma cells [9], and by upregulating the c-Jun N-terminal kinases (JNK) pathway in human osteosarcoma cells [12]. A recent study also demonstrated that curcumin-induced autophagy was mediated by the activation of the AMPK signaling pathway in human lung adenocarcinoma cells [19]. To determine the pathway that is responsible for the curcumin-induced expression of the hST8Sia I gene in A549 cells, we checked the promoter activity after treatment with specific inhibitors of these pathways. As shown in Figure 6, the promoter activity of the A549 cells harboring pGL3-1146/-646 was augmented in cells with curcumin treatment compared with untreated cells. The promoter activity enhanced by curcumin was not significantly inhibited by LY294002 (PI3K/AKT inhibitor), U0126 (MEK/ERK inhibitor), SP600125 (JNK inhibitor), and GO6983 (PKC inhibitor), whereas compound C (AMPK inhibitor) dramatically decreased curcumin-induced promoter activity. These data indicate that in curcumin-stimulated A549 cells, the transcriptional activation of the hST8Sia I gene is controlled by the AMPK signaling pathway.

## 3. Discussion

In the present study, immunofluorescence staining of LC3 showed that curcumin induced the autophagy in A549 cells. Our finding is in agreement with the findings in previous studies [19,20], demonstrating autophagy induction by curcumin in A549 cells. We also found that the gene expression of the hST8Sia I catalyzing ganglioside GD3 synthesis was significantly increased by the curcumin treatment in A549 cells. In addition, our data showed that the enhanced hST8Sia I gene expression by curcumin stimulation was correlated with a marked increase of ganglioside GD3 in curcumin-treated A549 cells, as demonstrated by immunostaining with anti-GD3 mAb.

In previous studies, we revealed that the hST8Sia I gene expression in human neuroblastoma SK-N-BE(2)-C cells was upregulated by valproic acid, a simple branched-chain fatty acid [30], and cordycepin, a naturally occurring adenosine analogue [32]. In contrast, the hST8Sia I gene expression was downregulated by the natural product triptolide in human melanoma SK-MEL-2 cells [31]. The transcriptional regulation of hST8Sia I gene in response to these substances was mediated by the NF-kB binding site located at -731 to -722 from the initial codon of the hST8Sia I gene [30,31,32]. Similarly, the result of the present study demonstrated that in A549 cells, curcumin upregulates the hST8Sia I gene transcription by activating NF-κB-mediated transcriptional activity in the hST8Sia I gene promoter, as evidenced by deletion analysis, site-directed mutagenesis, and in vivo ChIP assay.

NF-κB is a well-known transcription factor that is constitutively expressed in almost all cancer types. It regulates the expression of numerous genes involved in a wide variety of biological processes, including inflammation, immune and stress-induced responses, survival, apoptosis, and oncogenesis [33,34,35]. Recently, accumulating evidence indicates that curcumin downregulates the activation of the NF-κB signaling pathway and the expression of various oncogenes regulated by NF-κB [33,34,35,36,37,38,39,40,41]. In this study, however, our results clearly indicate that curcumin induces the activation of NF-κB, which results in the transcriptional activation of the hST8Sia I gene in A549 cells.

A previous study revealed that curcumin activated AMPK and subsequently inhibited the activation of NF-κB in human colon cancer cells, demonstrating that curcumin suppressed NF-κB via AMPK activation [42]. Pan et al. also reported that curcumin induces AMPK activation in ovarian cancer cells [43]. The present study clearly shows that curcumin triggered the transcriptional activation of the hST8Sia I gene via the AMPK signaling pathway in A549 cells, as demonstrated by the AMPK inhibitor. Our present finding is consistent with a previous study showing the occurrence of curcumin-induced autophagy via activating the AMPK signaling pathway in the A549 cells [19]. In contrast, while a previous finding demonstrated suppression of NF-κB via AMPK activation by curcumin in human colon cancer cells [42], our data indicate that in curcumin-stimulated A549 cells, the transcriptional activation of the hST8Sia I gene is induced by the activation of NF-κB via the AMPK signaling pathway. These results suggest that the suppression or activation of NF-κB via the AMPK signaling pathway by curcumin can vary depending on the types of cells.

Recently, Matarrese et al. demonstrated that in human primary fibroblasts, ganglioside GD3 plays a vital role in autolysosome formation and maturation through molecular interaction with the autophagy-related molecules LC3 and LAMP1 [27]. They also found that the siRNA-mediated knockdown of the hST8Sia I gene significantly inhibited autophagy [27]. These findings suggest that in curcumin-stimulated A549 cells, autophagy induction is caused by the upregulation of hST8Sia I gene expression.

## 4. Experimental Section

### 4.1. Cell Cultures

The human lung carcinoma cell line A549 obtained from Korean Cell Line Bank (KCLB, Seoul, Korea) was cultivated and maintained in RPMI-1640 media (WelGENE Co., Daegu, Korea) with 10% (*v/v*) heat-inactivated fetal bovine serum (FBS) (WelGENE Co., Daegu, Korea) and 1% penicillin-streptomycin (PS) solution (100 U/mL of penicillin, 100 μg/mL of streptomycin) at 37 °C in a 5% CO_2_ incubator. Curcumin purchased from Sigma-Aldrich (St. Louis. MO, USA) was dissolved in dimethyl sulfoxide (DMSO).

### 4.2. MTT Cell Viability Assay

To assess the viability of the cells, they were seeded in a 24-well plate (5 × 10^4^ cells /well) and grown for 24 h. The cells were exposed to different concentrations of MTT (0–80 μM) for 12 h and 24 h. MTT assay was carried out as described previously [30,31,32]. The cells were then reacted with the MTT assay mixture for 30 min. DMSO treatment was then performed to dissolve the reduced formazan crystal from MTT, and the absorbance at 540 nm was measured using the ELISA plate reader (Bio-rad, Hercules, CA, USA).

### 4.3. Reverse Transcription-Polymerase Chain Reaction (RT-PCR)

First-strand cDNA was synthesized using total RNA prepared from cultured cells with Trizol-Reagent (Invitrogen; Carlsbad, CA, USA) and the RNA to cDNA EcoDry^TM^ Premix (Oligo dT) kit (Clontech 639543). The resultant cDNA mixtures were amplified by PCR using the following primers: for hST8Sia I (460 bp), 5′-TGTGGTCCAGAAAGACATTTGTGGACA-3′ (forward) and 5′-TGGAGTGAGGTATCTTCACATGGGTCC-3′ (reverse); for β-actin (247 bp), 5′-CAAGAGATGGCCACGGCTGCT-3′ (forward) and 5′-TCCTTCTGCATCCTGTCGGCA-3′ (reverse). PCR amplification was performed using the same method as described previously [30,31,32].

### 4.4. Transfection and Luciferase Reporter Assays

The luciferase reporter plasmids used in this study, pGL3-1146/-646 to pGL3-2646/-646, have been reported elsewhere [30,31,32]. To assess the effect of curcumin on hST8Sia I promoter activity, the cells were cultivated in 24-well plates to about 60% confluency and transiently co-transfected with 0.5 μg of luciferase reporter plasmids and 50 ng of pRL-TK plasmid using Vivamagic (Vivagen Co., Gyunggido, Korea) according to the manufacturer’s protocols. Twenty-four hours after the transfection, the cells were treated for a further 24 h with 40 μM curcumin. The cells were then lysed and the Firefly and *Renilla* luciferase activities were evaluated using the Dual-luciferase Reporter Assay System (Promega, Madison, WI, USA) in accordance with the manufacturer’s instructions as well as a Glomax^TM^ 20/20 luminomerter (Promega, Madison, WI, USA). The Firefly luciferase activity of the reporter plasmid was normalized to the *Renilla* luciferase activity and expressed as a fold induction over the promoterless pGL3-basic vector as a negative control. Independent triplicate experiments were performed for each plasmid.

### 4.5. Immunofluorescence

Immunofluorescence staining was performed as previously described [44,45]. In brief, the cells were grown on sterile coverslips and treated with curcumin for 24 h. The cells were then fixed with 4% paraformaldehyde for 10 min at 37 °C, washed three times with phosphate-buffered saline (PBS), and blocked with 1% BSA for 1 h at 37 °C. In the LC3 experiment, after fixation and before blocking, the permeabilization step was performed with cold methanol for 10 min at −20 °C. The slides were incubated at 4 °C overnight with the anti-GD3 monoclonal antibody (mouse IgM, Kappa-chain, clone, GMR19; Seigakagu, Tokyo, Japan) or monoclonal anti-LC3B antibody (Cell Signaling Technology #3868S). After washing three times with PBS, the slides were incubated for 1 h at 37 °C with FITC-conjugated goat anti-rabbit IgG (Vector labs, F1-1000) as the secondary antibody. The nucleus was stained with 4′,6-diamidino-2-phenylindole (DAPI) at room temperature for 10 min. Fluorescence images were acquired using a LSM 700 confocal laser scanning microscope (Carl Zeiss, Oberkochen, Germany), as described in [46,47].

### 4.6. Chromatin Immunoprecipitation (ChIP) Assay

The ChIP assay was performed using a ChIP assay kit (Millipore, Billerica, MA, USA) according to the manufacturer’s instructions. Briefly, the cells were cross-linked in 1% formaldehyde at 37 °C for 5 min and then washed twice with ice-cold PBS containing Halt^TM^ protease inhibitor cocktail (Thermo Scientific, Rockford, IL, USA). The cells were then collected using a scraper and were lysed and sonicated to shear genomic DNAs to average size of 200–1000 bp. Immunoprecipitation was carried out using 4 μg of NF-κB antibody (Santa Cruz Biotechnology, Santa Cruz, CA, USA) and IgG antibody (Sigma) as the negative control. The cross-link was then reversed in the 5 M NaCl at 65 °C for 4 h and the remaining proteins were digested with proteinase K. DNA fragments were extracted with phenol-chloroform-isoamyl alcohol (25:24:1), precipitated with ethanol and then resuspended with water. Immunoprecipitated DNA was used for PCR analysis using primers surrounding the NF-κB binding site on the hST8Sia I promoter. Information on the hST8Sia I primer is as follows: (forward) 5′-CTCCGCCACACTCAGGGACT-3′ and (reverse) 5′-ACAAACGCCCGGGGATTG-3′.

## 5. Conclusions

In the present study, we demonstrated for the first time that curcumin induces upregulation of the hST8Sia I gene expression as well as autophagy in human lung adenocarcinoma A549 cells. In addition, we demonstrated that ganglioside GD3 production concomitant with hST8Sia I expression is also remarkably augmented in curcumin-stimulated A549. These results suggest that the curcumin-induced gene expression of hST8Sia I would simultaneously lead to GD3 biosynthesis in A549 cells. Furthermore, the current results indicated that the transcriptional activation of hST8Sia I gene was induced by the activation of the NF-κB via AMPK signaling pathway in curcumin-stimulated A549 cells.

## Abbreviations

AMPK	AMP-activated protein kinase
AP-1	Activator protein-1
BSA	Bovine serum albumin
ChIP	Chromatin immunoprecipitation
CREB	cAMP response element-binding protein
DAPI	4′,6-Diamidino-2-phenylindole
ERK	Extra cellular-signal-regulated kinase
FITC	Fluorescein isothiocyanate
IKK	IκB kinase
JNK	c-Jun N-terminal kinase
LAMP1	Lysosomal-associated membrane protein 1
LC3	Microtubule-associated protein 1 light chain 3
mAb	Monoclonal antibody
mTOR	Mechanistic target of rapamycin
NF-κB	Nuclear factor kappa B
PBS	Phosphate-buffered saline
PCR	Polymerase chain reaction
PI3K	Phosphatidylinositol 3-kinase
PKC	Protein kinase C
RPMI	Roswell Park Memorial Institute medium
